# Accumulation of heavy metals and antioxidant responses in *Vicia faba* plants grown on monometallic contaminated soil

**DOI:** 10.1007/s11356-012-1191-7

**Published:** 2012-09-23

**Authors:** Aleksandra Nadgórska-Socha, Alina Kafel, Marta Kandziora-Ciupa, Janina Gospodarek, Agnieszka Zawisza-Raszka

**Affiliations:** 1 Department of Ecology, University of Silesia, Bankowa 9, 40-007 Katowice, Poland; 2 Department of Animal Physiology and Ecotoxicology, University of Silesia, Bankowa 9, 40-007 Katowice, Poland; 3 Department of Agricultural Environment Protection, University of Agriculture in Krakow, Mickiewicza 21, 31-120 Kraków, Poland

**Keywords:** Antioxidant response, Metal contamination, *Vicia faba*

## Abstract

The purpose of this study was to explore the effects of soil contamination by selected metals (cadmium, copper, nickel, lead or zinc) on the antioxidant response of *Vicia faba* plants. The levels of the antioxidants: glutathione, proline, non-protein thiols, as well as guaiacol peroxidase and catalase activities were measured in the upperparts of plants. Additionally, the potential bioavailability of metals in the soil and their concentrations in *V. faba* plants were compared. Treatment with metal caused the problem of an elevation in its bioavailability in soil and its concentration in leaves and stems. The most serious problems seemed to be metal elevations in soil, especially Zn and Ni as well as in the aerial parts of *V. faba* plants. The antioxidant responses appeared to be metal specific. The elevation of guaiacol peroxidase activity in leaves and stems as well as the proline in leaves was the only more general reaction to metal exposure. Upon analysis of the effects of soil metal contamination on *V. faba* plants, we recommend the use of some measurements such as guaiacol peroxidase activity and proline level as useful tools in biological monitoring.

## Introduction

There are multiple sources of soil contamination with heavy metals such as industrial wastes, agriculture fertilizers and roadways. The problem of soil contamination may be of great importance for crops grown in the vicinity of heavy industrial sites. In such surroundings, the uptake of metals depends on the bioavailability of metals (Teklić et al. 2008). The bioavailable fraction of heavy metals is an issue of particular concern from ecological, toxicological and health standpoints, due to the possible penetration into most environmental segments, including food chains (Kucharski et al. 2005).

Heavy metals like cadmium (Cd), copper (Cu), nickel (Ni), lead (Pb), zinc (Zn) may affect the status of plants differently. Some of them are essential elements for cellular metabolism (Cu, Zn, Ni), while some are non-essential (Cd, Pb). Cu and Zn, are constituents of many enzymes and other proteins. The requirement of plants for Ni appears to be mainly related to its role in forming the active metallocentre of urease (Gratão et al. 2005; Page and Feller 2005).

The redistribution of metals within plants is metal specific (Page and Feller 2005). The elevation of non-essential metals like Pb, Cd and micronutrients such as Zn, Cu and Ni may be the cause of several negative aspects of oxidative stress (Zengin and Munzuroglu 2005; Meng et al. 2007). Therefore, the effectiveness of a plant’s antioxidant defense may be crucial for elucidating its tolerance mechanisms to heavy metals, which are common contaminants of soil. The synthesis of diverse metabolites in millimolar concentrations, particularly specific amino acids (such as proline and histidine) and peptides such as glutathione (GSH) or phytochelatins (PC), may be important for defense mechanisms against a metal’s action. Low molecular weight antioxidants such as proline, ascorbic acid or glutathione detoxify oxygen free radicals. Non-protein compounds, rich in –SH groups, are capable of binding metal ions and forming non-toxic complexes with metals. They are also involved in determining a plant’s tolerance to heavy metal ions, which is another important factor (Wei et al. 2003; Sharma and Dietz 2006; Sun et al. 2007; Xu et al. 2009). The accumulation of proline, a very effective singlet-oxygen quencher and redox active metal ions binder, activates and protects enzymes such as catalase (CAT) and guaiacol peroxidase (POD) (Teklić et al. 2008). The latter enzyme is involved in several processes such as cell growth, auxin catabolism, lignifications as well as abiotic and biotic stress responses (Fang and Kao 2000; Cui and Wang 2006).

Information about the response of particular cultivations to heavy metal contamination would be important in an assessment of the safe production of crops. On the other hand, some plant cultivation on metal-contaminated soil could be connected with the immobilization of metals by plants. In the experiment of Lopareva-Pohu et al. (2011), the bioavailability of metals as well as their uptake by two plants, *Lolium perenne* (Poaceae) and *Trifolium repens* (Fabaceae), was investigated. The examined plants were able to reduce the bioavailability of the elements. Probst et al. (2009) emphasized the important role of other leguminous representatives for instance *Vicia faba* plants, in cultivation on soil and metal tailings. They found that translocation was most significant for Zn and Cd but limited for Pb.

This study was undertaken in order to quantify the impact of different heavy metals applied to the soil on the antioxidant status of *V. faba* plants and to evaluate plant growth. The usefulness of these measurements as biomarkers of metal soil contamination was evaluated. To achieve this goal, we determined and compared the levels of metals in the leaves and stems of *V. faba* plants. We studied bioavailability of single metals, Cd, Cu, Ni, Pb and Zn, in the soils at the end of the experiment. We also determined the levels of antioxidants (non-protein thiols, GSHt, and proline) and antioxidant enzymes (guaiacol peroxidase and catalase) in order to complement chemical measurements.

## Materials and methods

### Soil

The field study area was in Zagaje Stradowskie (a rural village situated in the province of Świętokrzyskie, Poland), whose characteristics were given in Kafel et al. (2010). Broad bean plants (*V. faba* ssp. major) were cultivated in pots filled with soil contaminated with heavy metals. The soil pH was 5.3 in 1 M KCl, 6.1 in water and the soil contained 1.13 % of organic carbon. The soil was amended with heavy metals 1 month before cultivation and allowed to equilibrate. Each pot contained 9.8 kg of separately prepared soil. A thin layer of crumbled soil was put on plastic foil, sprayed with an appropriate mixture of fertilizer and metal and then soil was thoroughly mixed. This procedure was repeated several times. The following basic soil fertilization was applied—0.7 g N (as NH_4_NO_3_), 0.8 g P_2_O_5_ (as KH_2_PO_4_) and 1.2 K_2_O (as KCl) per one pot with a natural content of heavy metals (control group) and with soil contaminated with Pb, Cd, Cu, Ni and Zn. The heavy metals were added to the soil as a solution of Pb(NO_3_)_2_, CdSO_4_ x 8H_2_O, CuSO_4_, NiSO_4_ x 7H_2_O, ZnSO_4_ x 7H_2_O. The basis for the choice of metal concentrations in soil was the IUNG trace metals contamination level classification (III level—mean contamination). Limit values of heavy metal content in the topsoil were 5 mg kg^−1^ Cd, 100 mg kg^−1^ Cu, 1,000 mg kg^−1^ Pb, 1,500 mg kg^−1^ Zn, 150 mg kg^−1^ Ni. The heavy metal concentrations and the applied heavy metal doses in this study are presented in Table 1.

**Table 1:** Experimental groups according to heavy metal concentrations (mg∙kg^−1^ soil d.w.)

Experimental groups (heavy metals content)	Pb	Cd	Cu	Ni	Zn
Initial soil–control	28.2	0.6	8.2	12.8	52.9
Applied heavy metal doses	530	4	85	110	1,000

### Plant material

*V. faba* plants were cultivated for 2 months. Five plants were grown in each from ten pots. Summarized, each experimental group included 50 plants. Shoots 10, 30 and 80 cm in length (depending on metal treatment), and covered with leaves were collected randomly from the *V. faba* plants. The youngest, fully expanded, mature leaves and stems were used for the analyses. From each pot per treatment the plant material were mixed and each biochemical experiment was performed in five replicates. After a 2-month cultivation, the length between the apical leaf and basal stem was also measured and the shoot length and biomass of the whole aerial parts of plant were estimated. Each experimental group included twenty plants (from each pot two plants have been measured and weighted).

### Analysis of metal concentration in the soil and plants samples

The concentrations of the metals Pb, Cd, Cu, Ni, and Zn that were used for soil treatment, were analyzed in particular soil fractions and in the leaves and stems of plants. The soil metal content was estimated according to the method of Bouwman et al. (2001) and Ostrowska et al. (1991). Air-dried soil samples were sieved through a sieve with 1-mm pores and used for metal extraction with 0.01 M CaCl_2_ (potentially bioavailable elements) or with 2 M HNO_3_ (acid extracted elements). During CaCl_2_ extraction, 5 g of soil with 50 mL of a 0.01 M CaCl_2_ solution was shaken for 5 h. The HNO_3_-extractable fraction was obtained by shaking a sample (10 g) with 100 mL of 2 M HNO_3_ for 1 h. Finally, the content of metals was measured in the filtered extracts using flame absorption spectrometry (Unicam 939 Solar). The experiments were performed in five replicates.

In order to determine the heavy metal concentrations in the upper part of plants (leaves and stems), plant material was cleaned of any patches of deposited aphid honeydew and other surface contaminants, washed in tap, next in distilled water. It was then dried at 105 °C. A portion of 0.25 g dried plant material was digested with 5 mL of HNO_3_ at 110 °C and then diluted to 10 mL with deionized water. Next, the metal content (Zn, Pb, Ni, Cu and Cd) was measured using flame absorption spectrometry (Unicam 939 Solar). The quality of the analytical procedure was checked using a reference material (Certified Reference Material CTA-OTL-1 Oriental Tobacco Leaves) with the same quantities of samples. The mean recovery percentages of the reference material presented in milligrams of metal·per kilogram dry weight of sample was as followed 115 % of cadmium, 104 % of copper; 106 % of nickel, 110 % of lead and 99 % of zinc.

### Analysis of the biochemical parameters of the plants

Crushed plant parts were homogenized in a 100 mM phosphate buffer (pH 6.8) for the analysis of POD activity (1:7 ratio) and in a 50 mM (K/Na) phosphate buffer prior to CAT activity (1:5 ratio) and centrifuged at 12,000×*g* for 20 min. The supernatants were used to determine the enzyme activity levels. The whole procedure was carried out at 4 °C.

The guaiacol peroxidase activity was measured at 470 nm according to Fang and Kao (2000) with guaiacol as the substrate. The POD activity was measured in a reaction mixture (3 mL) containing a 50 mM phosphate buffer (pH 5.8), 1.6 μL H_2_O_2_, 1.5 μL guaiacol and 0.2 mL enzyme extract. The activity was calculated using the extinction coefficient (26 mM^−1^ cm^−1^) for tetra-guaiacol and was expressed in micromoles tetra-guaiacol per minute per milligram protein.

The catalase activity was determined by following the consumption of H_2_O_2_ (an extinction coefficient of 39.4 mM^−1^ cm^−1^) at 240 nm for 30 s (Aebi 1984) and was expressed in μmol consumed H_2_O_2_ min^−1^ mg protein^−1^. The reaction mixture (3 mL) contained a 50 mM potassium phosphate buffer (pH 7.0), 15 mM H_2_O_2_ and 0.2 mL enzyme extract.

To measure the contents of non-protein thiols, the plant material was homogenized in a 5 vol/g mixture containing 5-sulphosalicylic acid (2 g per 100 mL) and 1 mM EDTA and sodium ascorbate (0.15 g per 100 mL). The samples were centrifuged at 20,000×*g* for 10 min at 4 °C. Then a 0.5 mL liquid supernatant, 0.5 mL of a 1 M sodium phosphate buffer (pH 8.0) and 100 μl of 10 mM 5,5′-dithio-bis (2-nitrobenzoic acid) (DTNB) were put into test tubes. The absorbance at 415 nm was read 1 min after the addition of DTNB. The number of non-protein SH groups was established based on a curve prepared using l-cysteine and expressed as nanomoles –SH per gram fresh weight (Mass et al. 1987).

The acid–ninhydrin method was used to determine the proline content. The plant material (0.5 g) was homogenized in 10 mL of sulfosalicylic acid (3 g per 100 mL) and the homogenate was filtered through Whatman No. 2 filter paper. The reaction mixture containing 2 mL of homogenate, 2 mL of acid ninhydrin and 2 mL of glacial acetic acid was incubated at 100 °C for 1 h. The reaction mixture was placed on ice and extracted with 4 mL of toluene. The absorbance was read at 520 nm using toluene as the blank. The proline content expressed in micromoles proline per gram fresh weight was calculated as described by Bates et al. (1973).

The content of total glutathione (GSHt) were measured according to Anderson (1985) procedure and proteins content according to Bradford (1976) procedure using the appropriate standard curves of oxidized glutathione and bovine standard albumin solutions, respectively.

To detect the glutathione concentration, plant parts (0.5 g) were homogenized in TCA (trichloroacetic acid, 5 g per 100 mL) and a 0.125 mM phosphate buffer (pH 6.3) with 6.3 mM EDTA and were centrifuged at 10,000×*g* for 10 min at 4 °C. Supernatants were used for GSH determinations using the DTNB–oxidized glutathione reductase recycling procedure according to Anderson (1985). The reaction mixture contained 0.2 mL of supernatant, 0.6 mL of 0.3 mM NADPH, 0.1 mL of 6 mM DTNB and 0.1 mL (0.5 IU mL^−1^) of glutathione reductase (Sigma). The linear changes in the absorbance of the reaction mixtures were measured at 412 nm and the GSHt was expressed as μmol GSH g^−1^ fresh weight.

### Statistical assessment

The biochemical parameters data and metal content were analyzed, checked for normality and equality of variance, and when necessary the data were log transformed. The data were analyzed by ANOVA and the treatments were treated as the independent variables. Significant statistical differences of all variables were established using the Tukey test (ANOVA; Statistica 8 package). We also calculated the linear correlation coefficient between the metal concentrations in separate soil extractants and in the stems and leaves of *V. faba*, and also, between the metal concentrations and biochemical parameters in the aerial parts of *V. faba*.

## Results

### The bioavailability of metals in soil

Soil that was contaminated with metals (Pb, Cd, Cu, Ni, or Zn) was characterized by higher metal bioavailability in comparison with the control soil. Additionally, there was a clear difference between the concentration of metals in the fraction of soil extracted with HNO_3_ and the fraction of soil extracted with CaCl_2_. Among the metals examined, the highest concentrations of Pb and Zn were measured in the acid extracted fraction of the soil. A several times lower concentration of the metals examined were measured in the potentially bioavailable fraction (CaCl_2_ extracted). Potentially bioavailable Cu, Pb contents made up less than 1–3 % of the metal amounts that were HNO_3_ extracted (Tables 2 and 3). Compared with Zn amounts that were acid extracted, 40.2 % of the Zn was extracted with CaCl_2_. In addition, 27.5 % of the Cd was CaCl_2_ extracted compared with the Cd amounts that were acid extracted. However, compared to metal doses added to the soil, Cd was more extractable than Zn (Tables 1, 3, and 4). The CaCl_2_ fractions of Zn and Cd averaged from 14.8 to 35.5 % of the metal dose (respectively 1,000 and 4 mg kg^−1^) amended in the soil.

**Table 2:** The concentrations of selected metals in fractions of the soils (mg∙kg^−1^) extracted with HNO_3_

Contamination variant	Metal concentration				
	Pb	Cd	Cu	Ni	Zn
Pb	370.5 ± 20.5 c	2.5 ± 0.02 b	2.1 ± 0.3 a	0.9 ± 0.2 a	10.9 ± 0.5 a
Cd	5.1 ± 1.35 a	5.1 ± 0.2 b	1.6 ± 0.1 a	0.9 ± 0.1 a	7.5 ± 0.6 a
Cu	18.1 ± 4.9 b	2.4 ± 0.1 b	59.9 ± 0.8 b	0.6 ± 0.2 a	10.0 ± 0.9 a
Ni	6.7 ± 0.4 a	1.4 ± 0.1 ab	1.2 ± 0.1 a	34.5 ± 0.3 b	13.6 ± 0.4 a
Zn	8.5 ± 0.4 a	2.1 ± 0.13 ab	1.6 ± 0.2 a	0.8 ± 0.1 a	364.3 ± 3.5 b
C	6.0 ± 0.2 a	0.7 ± 0.2 a	1.9 ± 0.1 a	0.7 ± 0.2 a	8.6 ± 1.0 a

The different letters denote significant differences between the particular metal concentrations in the fraction extracted with HNO_3_ (*p* < 0.05)

*C* stands for control in all tables

**Table 3:** The concentrations of selected metals in fractions of the soils (mg∙kg^−1^) extracted with CaCl_2_

Contamination variant	Metal concentration				
	Pb	Cd	Cu	Ni	Zn
Pb	1.5 ± 0.03 b	0.3 ± 0.1 ab	nd	0.03 ± 0.01 a	1.5 ± 0.04 a
Cd	1.2 ± 0.1 b	1.4 ± 0.4 c	nd	0.04 ± 0.02 a	1.3 ± 0.3 a
Cu	0.3 ± 0.1 a	0.8 ± 0.03 b	1.3 ± 0.1 a	0.02 ± 0.02 a	1.6 ± 0.1 a
Ni	0.4 ± 0.4 a	0.3 ± 0.03 ab	nd	6.5 ± 1.6 b	1.8 ± 0.5 a
Zn	0.3 ± 0.02 a	0.5 ± 0.02 ab	0.1 ± 0.01 b	0.3 ± 0.2 a	146.5 ± 6.5 b
C	0.5 ± 0.1 a	0.1 ± 0.04 a	nd	0.2 ± 0.3 a	1.4 ± 0.4 a

The different letters denote significant differences between the particular metal concentrations in the fraction extracted with CaCl_2_ (*p* < 0.05)

**Table 4:** Heavy metal bioavailability comparison in contaminated soil samples (CaCl_2_ extraction)

Metal	Pb	Cd	Cu	Ni	Zn
Mean concentration in contaminated soil (mg∙kg^−1^ d.w.)	1.52	1.42	1.32	6.59	148.42
Availability in % (applied metal dose = 100 %)	0.29	35.46	1.55	5.99	14.84

The heavy metal concentrations in soil extracts were calculated on the basis of soil dry weight

### Heavy metal concentration in plants

The mean values of heavy metal concentrations in *V. faba* leaves and stems were found in a descending order—Zn > Ni > Pb > Cu > Cd. Generally, the increase in metal concentrations in the leaves and stems of the plants investigated in comparison to the control group was found in metal-contaminated soil (Table 5).We found a strong positive correlation between concentrations of all of the metals examined in separate soil extractants and in particular parts of the plants that were examined. The range of coefficients was 0.53–0.99 with *p* < 0.05. There was no correlation between the Cd concentration in stems and the Cd concentration measured in both extractants (Table 6).

**Table 5:** The concentrations of heavy metals (mg∙kg^−1^ d.w.) in the leaves and stems of *V. faba* plants grown in soil contaminated with heavy metals

Contamination variant	Metal concentration				
	Pb	Cd	Cu	Ni	Zn
Leaves					
Pb	20.41 ± 2.78^a,b^	0.38 ± 0.15	0.62 ± 0.16^a,b^	3.43 ± 0.32^a^	51.86 ± 6.51^b^
Cd	4.34 ± 0. 34^a,b^	1.58 ± 0.06^a,b^	2.17 ± 0.32^a^	3.88 ± 0.80^a^	50.42 ± 3.92^b^
Cu	0.85 ± 0.11^a,b^	0.24 ± 0.09	3.53 ± 0.35^b^	5.43 ± 0.17^a,b^	22.30 ± 0.63^a,b^
Ni	0.27 ± 0.04^a^	0.10 ± 0.04^b^	2.78 ± 0.28^b^	85.42 ± 8.69^a,b^	58.50 ± 1.57^a,b^
Zn	0.69 ± 0.14^a,b^	0.14 ± 0.02^a^	3.30 ± 0.25^b^	8.60 ± 1.33^b^	1748.50 ± 24.15^a,b^
C	0.19 ± 0.06^a^	0.09 ± 0.05	1.85 ± 0.03	3.87 ± 0.42^a^	34.74 ± 2.38^a^
Stems					
Pb	16.35 ± 0.72^a,b^	0.21 ± 0.10	1.57 ± 0.21^a^	9.83 ± 0.59^a,b^	58.41 ± 1.77^b^
Cd	1.29 ± 0.06^a^	0.62 ± 0.07^a,b^	1.48 ± 0.22^a^	7.21 ± 0.24^a^	48.47 ± 6.10^b^
Cu	0.58 ± 0.08^a,b^	0.35 ± 0.04^b^	3.83 ± 0.22^b^	6.76 ± 0.41^a^	27.60 ± 1.72^a,b^
Ni	0.09 ± 0.05^a,b^	0.13 ± 0.02	2.82 ± 0.20^b^	50.07 ± 3.92^a,b^	19.83 ± 1.64^a,b^
Zn	2.12 ± 0.13^a,b^	0.40 ± 0.05^a,b^	3.38 ± 0.21^b^	10.65 ± 0.44^b^	1211.25 ± 131.04^a,b^
C	1.23 ± 0.17^a^	0.15 ± 0.03	1.87 ± 0.08	6.82 ± 0.42^a^	64.90 ± 2.50^a^

^a^Denotes significant differences between the metal concentration in leaves and stems in one variant of contamination

^b^Denotes significant differences between the metal concentration in plants grown in the contaminated soil and the control (*p* < 0.05)

**Table 6:** The correlation coefficients between the concentrations of particular metals in separate soil extractants (with HNO_3_ or CaCl_2_) and in the leaves and stems of *V. faba* plants (*p* < 0.05)

	Pb		Cd		Cu		Ni		Zn	
	HNO_3_	CaCl_2_	HNO_3_	CaCl_2_	HNO_3_	CaCl_2_	HNO_3_	CaCl_2_	HNO_3_	CaCl_2_
Leaves	0.97	0.81	0.92	0.84	0.53	0.59	0.99	0.97	0.90	0.90
Stems	0.99	0.72	NS	NS	0.60	0.66	0.99	0.97	0.98	0.90

*NS* not significant

### The biomass and shoot length

**Fig. 1:**
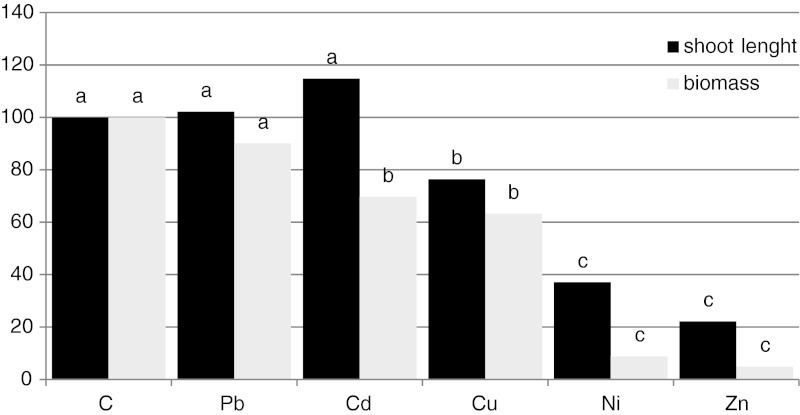
The mean shoot length and weight of an aerial part per plant (in % of control) of *V. faba* plants cultivated in the control and in soil contaminated with individual heavy metal. Control features constituted 100 %

The highest decrease in mean shoot length of *V. faba* plants was found in plants cultivated on soil contaminated with Zn and Ni and the decrease was observed also in plants cultivated on soil contaminated with Cu. These metals and Cd contaminations also caused a reduction in biomass. The highest reduction in plant aerial parts biomass was observed for Zn- (almost a 95 % decrease in comparison to the control plants) and for Ni-treated plants (a 91 % decrease; Fig. 1).

### The biochemical status of the plants

For Zn-treated plant the increase in glutathione was found in leaves, while in the case of Cu- and Ni-treated plants it was recorded in stems. In the case of Pb treatment, an elevation was measured in both plant parts. The highest GSHt concentration (79.4 μmol GSH g^−1^ fresh weight) was recorded in the leaves of Zn-treated plants, more than 2.6 times than in the control plants (Table 7). The total glutathione pool level was positively correlated with Zn content in leaves. In addition, a positive correlation was found between Pb, Ni, and Cu and glutathione concentration in stems of *V. faba* (Table 8). Generally, a higher GSHt pool was found in stems than in the leaves (cases of Pb, Cd, Cu, and Ni soil treatments). An opposite tendency was recorded for non-protein thiols when their concentrations in leaves and stems were compared. Moreover, their concentration was mostly lower in the leaves of plants that were exposed to metals compared with the controls. In plant stems treated with Cd and Zn, a decrease in the concentrations of non-protein thiols was also measured. A higher concentration of non-protein -SH groups in stems of *V. faba* plants treated with Pb, Cu, Ni than in stems of control plants was recorded. It ranged from 89.1 to 205.1 nmol –SH g^−1^ fresh weight in this part of the plants (Table 7). Finally, some correlations between non-protein –SH groups and metal concentrations in the leaves of examined plants were found, namely a negative relationship in cases of Pb or Cd, and a positive one in the case of Ni concentration (Table 8).

**Table 7:** Antioxidant concentrations (nmol –SH g^−1^ fresh weight, μmol GSH g^−1^ fresh weight, μmol proline g^−1^ fresh weight) in the leaves and stems of *V. faba* plants grown on soil contaminated with heavy metals

	Glutathione		Non-protein –SH groups		Proline	
	Leaves	Stems	Leaves	Stems	Leaves	Stems
Pb	38.1 ± 0.7^a,b^	42.3 ± 2.1^a,b^	270.4 ± 16.7^a,b^	145.9 ± 6.8^a,b^	0.8 ± 0.06^a,b^	0.4 ± 0.02^a^
Cd	16.4 ± 0.4^a^	29.6 ± 0.3^a^	247.2 ± 2.0^a,b^	89.1 ± 3.6^a,b^	0.8 ± 0.03^a,b^	0.4 ± 0.02^a^
Cu	16.7 ± 1.2^a,b^	40.1 ± 0.9^a,b^	290.8 ± 5.5^a^	205.1 ± 12.4^a,b^	0.6 ± 0.02^a^	0.2 ± 0.02^a^
Ni	30.3 ± 0.9^a^	42.1 ± 0.5^a,b^	311.4 ± 14.2^a^	147.7 ± 4.4^a,b^	2.7 ± 0.04^a,b^	2.2 ± 0.02^a,b^
Zn	79.4 ± 7.8^a,b^	36.4 ± 0.9^a^	278.1 ± 11.1^a,b^	93.0 ± 4.0^a,b^	3.2 ± 0.01^a,b^	2.2 ± 0.02^a,b^
C	29.5 ± 1.5	31.9 ± 3.1	309.8 ± 13.5^a^	123.3 ± 8.5^a^	0.5 ± 0.02	0.3 ± 0.01

^a^Denotes significant differences between the antioxidant concentrations in the leaves and stems data in one variant of contamination

^b^Denotes significant differences between the heavy metal concentrations in plants grown in the contaminated soil and the control (*p* < 0.05)

**Table 8:** The correlation coefficients between metal concentration and antioxidant measurements in the leaves and stems of *V. faba* plants (*p* < 0.05)

	Pb		Cd		Cu		Ni		Zn	
	Leaves	Stems	Leaves	Stems	Leaves	Stems	Leaves	Stems	Leaves	Stems
Glutathione concentrations	NS	0.38	−0.41	NS	NS	0.41	NS	0.48	0.92	NS
Non-protein thiols	−0.37	NS	−0.76	NS	NS	NS	0.45	NS	NS	NS
Proline concentrations	NS	NS	NS	NS	0.42	0.45	0.55	0.68	0.74	0.61
Peroxidase level	NS	NS	NS	NS	NS	NS	NS	0.47	0.81	0.59
Catalase level	NS	NS	−0.51	NS	NS	0.53	0.71	NS	NS	0.95

*NS* not significant

We detected an increase of proline concentration in the leaves of *V. faba* plants in most cases of metal treatment, as well as in the stems of plants grown on Zn and Ni contaminated soil. The concentration of this amino acid was higher in the leaves than in the stems of all plants that were exposed to metals, whereas the control plants had comparable amounts of this amino acid in both organs (Table 7). The proline content positively correlated with Zn, Ni, and Cu concentrations in the upper parts (leaves and stems of plants) (Table 8).

The activities of the enzymes CAT and POD were much higher in the stems than in the leaves of the plants examined. General, POD activity was higher in metal stressed plants than in the control. The activity of POD ranged from 93.5 to 182.4 μmol tetra-guaiacol min^−1^ mg protein^−1^ in leaves and 97.6–236.3 μmol tetra-guaiacol min^−1^ mg protein^−1^ in stems. CAT activity was higher in cases of Ni treatment (in both aerial parts of plants) and Cu or Zn treatments (but only in the stems of plants) in comparison to the control (Table 9). Positive correlations were found between CAT activity and Ni concentration in the leaves and Zn, Cu concentrations in the stems of plants. A negative correlation was found between the Cd concentration and CAT activity in leaves. In addition, POD activity positively correlated with Zn content measured in both parts of the plants and with Ni content in stems (Table 8).

**Table 9:** Antioxidant enzyme activity (respectively, μmol tetra-guaiacol min^−1^ mg protein^−1^, μmol consumed H_2_O_2_ min^−1^ mg protein^−1^) in the leaves and stems of *V. faba* plants grown on soil contaminated with heavy metals

	Guaiacol peroxidase		Catalase	
	Leaves	Stems	Leaves	Stems
Pb	117.09 ± 3.83^a,b^	185.12 ± 17.4^a,b^	71.6 ± 5.5^a^	234.0 ± 32.2^a^
Cd	108.29 ± 2.37^a,b^	171.47 ± 14.02^a,b^	36.8 ± 8.7^a,b^	207.1 ± 41.0^a^
Cu	83.52 ± 1.59^a,b^	126.17 ± 6.8^a,b^	35.0 ± 2.5^a,b^	265.7 ± 39.7^a,b^
Ni	128.57 ± 17.82^a,b^	217.12 ± 3.35^a,b^	99.7 ± 17.5^a,b^	273.0 ± 27.1^a,b^
Zn	182.39 ± 4.45^a,b^	236.34 ± 6.05^a,b^	75.1 ± 9.4^a^	708.1 ± 118.6^a,b^
C	93.50 ± 3.21	97.63 ± 2.95	58.4 ± 11.1^a^	187.5 ± 70.9^a^

^a^Denotes significant differences between the enzyme activity in the data for leaves and stems in one variant of contamination

^b^Denotes significant differences between the enzyme level in plants grown in the contaminated soil and the control (*p* < 0.05)

## Discussion

Whether treatment with metals increased their bioavailability in soil was evaluated using two metal extractions with CaCl_2_ and HNO_3_. The most available among the metals examined were Cd and Zn (Tables 3 and 4).

However, the significant differences in the bioavailability among the examined metals depending on CaCl_2_ and HNO_3_ extraction should be mentioned. Banjoko and McGrath (1991) found that CaCl_2_ extractable metal content is much more suitable than total metal content in predicting the bioavailability of heavy metals. Evaluations of the pool of soluble—potentially bioavailable trace elements in soil—are mainly based on different extractions done with various solutions, among which are acids or neutral salts such as CaCl_2_, NaNO_3_, NH_4_OAc (Kabata-Pendias 2004; Feng et al. 2005). Menzies et al. (2007) found that salts such as 0.01 M CaCl_2_ and 0.1 M NaNO_3_ provided the most useful indication of metal phytobioavailability across a range of metals. Pongrac et al. (2009) showed that ammonium acetate (NH_4_OAc) extraction increased Cd bioavailability (11.3–37.2 %). Cd extractability was similar to our results obtained after CaCl_2_ extraction.

Jiang et al. (2010) found that NH_4_OAc-extractable Zn and Cd concentrations were significantly reduced in multiple metal-contaminated soils planted with the Zn and Cd hyperaccumulator *Sedum plumbizincicola* in comparison with unplanted soils.

Plants, as important components of the ecological system, transfer metals from abiotic to biotic environments. The metal fraction in the soil that interacts with a biological target determines its bioavailability (Chojnacka et al. 2005; Mishra et al. 2009a). In our experiments, some differences in metal concentration series order in the soil extracts and the upper parts of *V. faba* plants were found. The metal concentrations in the soil fraction extracted with HNO_3_ decreased as follows: Pb = Zn > Cu > Ni > Cd and for the CaCl_2_ extracts decreased with order Zn > Ni > Pb = Cu = Cd. In general, the metal concentrations series order in stems and leaves was similar as in CaCl_2_ extractants—Zn > Ni > Pb > Cu > Cd. Similar trends were also observed by Celik et al. (2005) in the case of *Robinia pseudoacacia*. The metal concentrations measured in *R. pseudoacacia* was in the order of Fe > Mn > Zn > Pb > Cu > Cd. The increase of Zn, Ni, Pb and Cu in the upperparts of plants cultivated in our experiments was concomitant with an increase in metal concentrations in the fractions of soil extracted with CaCl_2_ and HNO_3_.

In this study, we found a higher concentration of metals (except Cu) in the leaves than in the stems of plants on metal contaminated soil. Similar results were obtained by Probst et al. (2009) during a cultivation experiment using *V. faba*, in which metal concentrations in plant roots, leaves and shoots were measured. Metal concentration—and generally bioaccumulation—was in the following order: roots > leaves > stems, except of Pb and Cd. In our study, the highest amounts of Zn and Ni among examined metals were accumulated in the leaves and stems of *V. faba* plants and this caused the most toxic effects on plant growth (Table 5).

Generally, toxic concentrations of Cd and Pb for plants are defined in ranges of 5–30 and 30–300 mg kg^−1^ d.w., respectively. Toxicity limits for Zn are in the range of 300–400 mg kg^−1^ d.w., depending on the plant species and the growth stage. Ni is readily taken up from soils by plants and its phytotoxic concentration range is generally 40–246 mg kg^−1^ d.w., whereas toxicity limits for Cu were established in the range of 20–100 mg kg^−1^ d.w. (Alloway and Ayres 1999; Kabata-Pendias 2001 ). In our investigation, the measured concentrations of Pb (20.4; 16.3 mg kg^−1^ in leaves and stems respectively), Cd (1.6; 0.6 mg kg^−1^) and Cu (3.5; 3.8 mg kg^−1^) in the aboveground parts of *V. faba* were below the toxic threshold. The Zn concentration in plants grown in soil amended with this metal (1211.3–1748.5 mg kg^−1^) was much higher than the established toxic threshold. The Ni concentration detected in leaves and stems (85.4; 50.1 mg kg^−1^) was within the toxic range for this metal. The toxicity caused by heavy metals is a concern because it inhibits plant growth thus leading to a lower yield; it also causes a deterioration in crop quality (Guo et al. 2004). In our experiment, we found that plant shoot lengths decreased (except for Cd- and Pb-treated plants) and that there was a reduction in biomass (except for Pb-treated plants) in comparison to the control plants. The results of Wang et al. (2010) showed that soil Pb can cause phytotoxicity to *V. faba* seedlings, which was evidenced by the significant decrease in shoot heights but only at higher concentrations of 1,000–2,000 mg kg^−1^. Similar to our results, soil Pb in concentrations of 500 mg kg^−1^and lower Pb doses did not cause shoot height to decrease. Kasim (2005) also found a decrease in *V. faba* shoot height and leaflet area as well as a decrease in the fresh and dry weights caused by heavy metals in following order Cu > Cu + Cd > Cd. The negative effect of metals on *V. faba* plant growth was also described by Probst et al. (2009). They observed reduced *V. faba* growth caused by high concentrations of metals, especially Zn, Pb, Mn in the mine tailings in which *V. faba* plants were cultivated. The growth of *V. faba* seedlings grown on mine tailings was found to be restrained by 38 % as compared to the control group. The stratagems of *V. faba* undergoing high concentrations of toxic metals in a carbonate substrate suggest root cell wall thickening in order to decrease the uptake of toxic metals. Cell wall thickening, which was observed by Probst et al. (2009), seems to be associated with an increased activity of peroxidase. This enzyme is able to catalyze the lignin synthesis and is induced in higher plants that are exposed to toxic metals (Liu at al. 2004; Gill and Tuteja 2010).

In our experiment, a variation in the antioxidant level, which was dependent on metal treatment, was noticed. In addition to enzymatic antioxidants, non-enzymatic antioxidants are important in heavy metals plants defense (Gill and Tuteja 2010).

An increase in GSH in the leaves of plants cultivated in soil contaminated with Pb and Zn was found. Zn content in *V. faba* leaves as well as glutathione content in this part of the plant positively correlated with the available Zn in soil (correlation coefficient 0.92). Similar results were obtained by Wang et al. (2010). Nocito et al. (2006) found that Cd and Zn affected the GSHt content of roots in different ways. An increase of the glutathione pool was noticed in the leaves of *Philadelphus coronarius* grown on a polluted site (Kafel et al. 2010). Treatment of *R. pseudoacacia* with Pb in investigations by Wińska-Krysiak and Bernat (2008) showed that Pb at a dose of 45 mg Pb^2+^ mg^−1^ in a hydroponic culture did not affect the GSHt level in roots, while in leaves the metal at this concentration led to an increase that was twice as high in comparison with the control plants. This can be attributed to the high demand of plants for GSH due to the synthesis of phytochelatins in response to a high accumulation of Pb in these organs.

Glutathione creates complexes with heavy metals and an induction of glutathione as well as cysteine synthesis has been documented in plants as a response to heavy metals stress (Arya et al. 2008). The changes in GSHt level are dependent on the metal treatment and the part of plant. The level of glutathione increases significantly under most metal treatment, at least, in one of the examined plant parts (stems or leaves). In this work, this level was positively related with concentration of metals (Pb, Cu, Ni) in stems (Tables 7 and 8) most of the time. It is interesting that in the case of Zn (which exhibits significant effects on plant growth, which was mentioned above), we found a positive correlation between GSHt and the Zn concentration in the leaves of *V. faba.* In contrast to our findings for two varieties of *Abelmoschus esculentus* exposed to mine spoil, an increase of GSH was measured mainly in the leaves rather than in other organs, where the level of this tripeptide declined (Arya et al. 2008). Arya and Roy (2011) found that in Mn-treated *V. faba*, the GSHt content was higher in the roots than in the stems. Moreover, the increase of total glutathione content was positively related to an increase in the Mn concentration in the roots and stems along with the duration of experiment. The total glutathione content initially increased up to 40 μM Mn and then declined with an increase in Mn concentrations. El-Beltagi and Mohamed (2010) found that an increase in Pb accumulation in *Raphanus sativus* leaves and roots was correlated with increasing metal concentration exposure to plants, although a decreasing trend in glutathione level was observed.

Glutathione might play a key role among non-protein thiols in a defense against a Zn surplus in the case of *V. faba* plants. In addition to a higher GSHt pool, a decrease in non-protein –SH groups was registered in Zn-treated plants in our study (Table 7).

A tolerance to metals may be due to changes in the content of non-protein thiols, which includes glutathione thiol-rich peptides (known as phytochelatins) or other –SH rich compounds. Pro-oxidant compounds, such as H_2_O_2,_ can selectively oxidize thiols. Molecules containing sulfur, which exist in a wide variety in cells, may fulfill different functions and may be independently regulated (Mishra et al. 2009b). In our study, the content of non-protein thiols decreased in the leaves and stems in Cd- and Zn-treated *V. faba* plants. This was similar to the study of De la Rosa et al. (2005). The production of thiols decreased in the leaves of *Salsola kali* at the highest tested concentration of Cd.

Moreover, in the case of Cu and Ni exposure in our study, apart from GSH, the role of non-protein thiols might be important in a defense response against an elevation in metals. We found a significant elevation of GSH and non-protein thiols in *V. faba* stems.

Most of the general responses of *V. faba* plants to different metals appear to be connected with the participation of peroxidase and proline. In our investigation, an increase of peroxidase activity in stems and leaves (except for Cu-treated plants) and proline concentration in leaves was established among plants treated with different metals. A similar effect, an elevated proline level, was measured in leaves of cabbage plants treated with Co, Ni, and Cd (Teklić et al. 2008). Verma and Dubey (2003) observed an increase of peroxidase in the stems and roots of rice cultivars after Pb treatment; however, the increase of the proline content was specific to plants from different experimental groups. In our experiment, the highest proline content was found in both aboveground parts (leaves and stems) of *V. faba* plants grown in soil contaminated with Zn or Ni. Proline accumulation is regarded as an indicator of environmental stress. Upregulation of proline under heavy metal stress conditions is often found in plants ranging from algae to angiosperms. A higher level of this amino acid increases the stress tolerance of plants through such mechanisms as osmoregulation, protection of enzymes against denaturation or stabilization of protein synthesis (Schat et al. 1997; Zengin and Munzuroglu 2005; Sharma and Dietz 2006; Xu et al. 2009). Sharma and Dietz (2006) listed several examples of flowering plants (*Cajanus cajan*, *Vigna mung*, *Helianthus annus*, *Lemna minor*, *Triticum aestivum*, *Lactuca sativa*, *Silene vulgaris*, *Oryza sativa*) that respond to heavy metals through an increase in proline level. In a field study, Kafel et al. (2010) registered an increase in proline level in the aboveground parts of *P. coronarius* in conditions of heavy metal traffic contamination. Zengin and Munzuroglu (2005) detected a significant increase of proline content along with ascorbic acid and α-tocopherol in bean leaves grown in a solution spiked with various concentrations of Pb, Cu, Cd and Hg.

POD enhancement was registered in the leaves of plants (except Cu) as well as in the stems (all metal treatments). The experiments of Hassan et al. (2008) and Shamsi et al. (2008) also showed POD enhancement in conditions of metal contamination in soybean plants exposed to Cd and in rice plants exposed to Cd in a hydroponic experiment. Elevated superoxide dismutase (SOD) and POD activities in the leaves and roots of barley along with an accumulation of Al, Cd and Cu were estimated by Guo et al. (2007).

Guaiacol peroxidase can be induced by heavy metals and is more efficient than CAT in eliminating H_2_O_2_ (Wang et al. 2010). It must be mentioned that the enhancement of antioxidant enzymes may be due to the upregulation of their genes expression. Antioxidant enzymes like SOD, CAT and ascorbate peroxidase upregulation are implicated in combating the oxidative stress caused by biotic and abiotic stress (including heavy metals) (Gill and Tuteja 2010). But the upregulation response may depend on the metal concentration as in the example of *Lepidium sativum*, where the upregulation of the enzymes took place in the presence of 100 and 200 mg kg ^−1^ Pb^2+^ representing a lower and moderate stress, while in the presence of higher concentrations, elevated expression was detected only at 400 mg kg^−1^ for CAT and ascorbate peroxidase.

Peroxidases, which are stress enzymes in plants, were used as a potential biomarker for sublethal toxicity in spruce seedlings (Radotić et al. 2000). Wang et al. (2010) found that for Pb-treated *V. faba* plants, POD enhancement and ascorbate peroxidase activity might be employed as an intrinsic and major defense tool responsible for H_2_O_2_ degradation under higher concentrations of Pb, in contrast to CAT, whose activity was reduced.

The elevation of CAT activity in Ni- or Zn-treated plants was noted in our study. Kafel et al. (2010) showed a similar phenomenon—an increase of CAT activity in the aboveground parts of *P. coronarius* grown in conditions of environmental pollution. In a pot experiment, Lin et al. (2007) showed that in *V. faba* exposed to Cd at 5 μg mL^−1^, the activities of POD, CAT, and SOD were significantly decreased, thus leading to an accumulation of reactive oxygen species.

POD activity enhancement in the leaves and stems of *V. faba* as well as proline accumulation in plants treated with Pb, Zn and Ni seem to be promising in investigations of a plant’s defense tools against the oxidative stress caused by heavy metals. A higher level of antioxidant defense was recorded in *V. faba* leaves and stems treated with Zn and Ni in comparison to plants treated with other metals. These results are related to the bioavailability of these metals and their higher accumulation within the plants.

## Conclusions

The general markers of different metal treatments in our experiment were POD activity and proline content. These parameters seem to be universal antioxidant defense factors against heavy metal exposure. The levels of Zn, Ni, Pb, Cd, and Cu, especially in the leaves of *V. faba*, reflected a contamination of the soil with these metals. Zn and Ni, in the doses investigated, are toxic to *V. faba* and inhibited plant growth (biomass and reduction in shoot lengths). Application of Zn and Ni led to a significant increase in these metals concentration detected in plants, which probably forced them to invest more energy into anti-oxidative defense. We found several examples of high correlations between antioxidant parameters and concentrations of metals in the upper parts of plants, higher POD and CAT activity as well as proline content, and in the case of Zn a higher GSHt content. The bioavailability of single metals and plant heavy metal response in particular cultivation investigations are recommended for the safe production of crops after contamination.

## Abbreviations

CAT: Catalase
GSHt: Glutathione total
POD: Guaiacol peroxidise
